# Quantification and kinetics of viral RNA transcripts produced in *Orthohantavirus* infected cells

**DOI:** 10.1186/s12985-018-0932-8

**Published:** 2018-01-19

**Authors:** Julia Wigren Byström, Jonas Näslund, Fredrik Trulsson, Magnus Evander, Olivia Wesula Lwande, Clas Ahlm, Göran Bucht

**Affiliations:** 1 Department of Clinical Microbiology, Infectious Diseases, Umeå University, Umeå, Sweden; 2 Swedish Defence Research Agency, CBRN Defence and Security, Umeå, Sweden; 3 Department of Clinical Microbiology, Virology, Umeå University, Umeå, Sweden

**Keywords:** *Orthohantavirus*, RNA segments, In-vitro infection, Quantitative real-time PCR

## Abstract

**Background:**

Rodent borne viruses of the *Orthohantavirus* genus cause hemorrhagic fever with renal syndrome among people in Eurasia, and hantavirus cardiopulmonary syndrome in the Americas. At present, there are no specific treatments or efficient vaccines against these diseases. Improved understanding of viral transcription and replication may instigate targeted treatment of *Orthohantavirus* infections. For this purpose, we investigated the kinetics and levels of viral RNA transcription during an ongoing infection in-vitro.

**Methods:**

Vero E6 cells were infected with Puumala *Orthohantavirus* (strain Kazan) before cells and supernatants were collected at different time points post infection for the detection of viral RNAs. A plasmid containing primer binding sites of the three *Orthohantavirus* segments small (S), medium (M) and large (L) was constructed and standard curves were generated to calculate the copy numbers of the individual transcripts in the collected samples.

**Results:**

Our results indicated a rapid increase in the copy number of viral RNAs after 9 h post infection. At peak days, 2–6 days after infection, the S- and M-segment transcripts became thousand and hundred-fold more abundant than the copy number of the L-segment RNA, respectively. The presence of viral RNA in the cell culture media was detected at later time-points.

**Conclusions:**

We have developed a method to follow RNA transcription in-vitro after synchronous infection of Vero cells. The obtained results may contribute to the understanding of the viral replication, and may have implications in the development of antiviral drugs targeting transcription or replication of negative stranded RNA viruses.

**Electronic supplementary material:**

The online version of this article (10.1186/s12985-018-0932-8) contains supplementary material, which is available to authorized users.

## Background

*Orthohantavirus* genus in the *hantaviridae* family comprises more than 20 rodent borne viruses pathogenic to humans. Rodents, bats, moles and shrews have been recognised to carry distinct members of the *Orthohantavirus* genus [1]. The geographical distribution of the various strains of these viruses follow the distribution of the respective animal species [2]. Humans acquire infection via inhalation or direct contact with rodent faeces, urine and saliva. Globally, 150–200,000 persons are infected each year [3, 4]. The virus infects the endothelium of various organs with symptoms of disease mainly from the kidneys and lungs [4]. Consequently, *Orthohantavirus* infections may lead to hemorrhagic fever with renal syndrome (HFRS) in Europe and Asia, or to the more lethal hantavirus cardiopulmonary syndrome (HCPS) in the Americas. The severe forms of HFRS has a case fatality rate of up to 12%, whereas the milder forms vary between 0.08% to 0.4% [5]. In Europe, more than 10,000 cases of HFRS are diagnosed annually, with increasing numbers, most of these cases are caused by Puumala *Orthohantavirus* (PUUV) [5]. HFRS caused by PUUV infection is characterized by symptoms such as fever, headache and abdominal pain with cardiovascular and gastrointestinal involvement [6, 7]. There are no efficient vaccines or specific treatments available against *Orthohantavirus* infections, which is highly needed due to the emergence of these potentially life-threatening diseases. To facilitate development of antivirals against *Orthohantavirus*es, a detailed understanding of the viral replication is required.

**Fig. 1:**
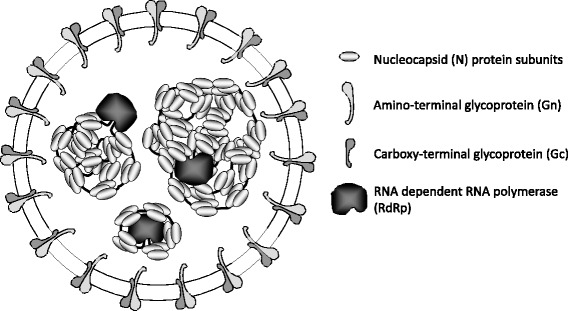
Schematic picture of a *Orthohantavirus* particle. The figure show the N protein covering the viral RNA, and the large RNA dependent RNA polymerase (RdRp). The spikes at the surface of the virus particle are heterodimers of the glycoproteins (Gn and Gc)

*Orthohantavirus* genomes are tri-segmented negative single-stranded RNA consisting of the Small (S), Medium (M) and Large (L) RNA segments [8, 9]. The S-segment encodes the nucleocapsid (N) protein consisting of 429–433 amino acid residues (about 50 kDa in size) and appears to be conserved between different *Orthohantavirus*es. The N protein plays a pivotal role in the life cycle of the virus and is the major antigen for the immune response, and abundantly expressed during the early phases of infection [2, 10]. The N protein coats the viral RNAs and protects it from degradation. All viral RNA segments are coated with the N protein by forming ribonucleoproteins (RNPs), thus establishing a favourable environment for replication, transcription and virus assembly. The RNPs are subsequently enclosed by an envelope consisting of a lipid bilayer with the surface glycoproteins embedded. This viral envelope is derived from the host cell membrane during the budding process of the virus. Each functional virion contains genomic RNA of the three segments, with molecules of the viral RNA-dependent RNA polymerase (RdRp) being attached to the viral RNA segments (Fig. 1). However, the generation and isolation of reassortant clones, and unstable diploid genomes, have been produced and isolated in-vitro [11, 12].

The M-segment encodes a polyprotein that is cleaved post translation into the glycoproteins Gn and Gc (N- and C-terminal portions of the polyprotein). These proteins, about 73 kDa and 56 kDa in size, are found at the surface of the cell and acquired by budding of viral ribonucleocapsids through the cellular membrane. This step enables enveloped viruses to cross the membrane barrier of cells. In a similar manner, the viral envelope permit viruses to enter cells discreetly through receptor attachment and virus-cell membrane fusion. Following endocytosis, these enveloped RNA viruses are directed to the endosomal compartment where a pH-dependent conformational change of the viral envelope glycoproteins mediate the fusion between the viral and endosomal membranes. Hence, viral envelopes allow virus assembly and disassembly during cell exit and entry, respectively. This striking duality is crucial for virus cell-to-cell transmission and dissemination within hosts [9, 13–15].

The L-segment is in average the most conserved part of the genome sequence of *Orthohantavirus* and encodes the RNA-dependent RNA polymerase (RdRp), the L protein (~ 240 kDa) [16]. This viral polymerase, possess transcriptase, replicase and endonuclease functions, but lacks the capacity to synthesize 5′cap sequences [9, 13, 14]. Similar to RdRp of related viruses, *Orthohantavirus* L protein acquires 5′cap sequence from cellular mRNA transcripts by a mechanism called “cap snatching”. The resulting short oligonucleotides containing the 5′cap is then used by the RdRp as a primer for synthesis of viral transcripts. While translation of mRNA requires a cap primer to allow translation of viral proteins, replication copies of the entire template does not depend on primers for initiation.

In this paper, we have investigated the kinetics and levels of viral RNA encoded from the three different segments at different time-points after synchronous infection of cells. Our findings may provide new insights into the *Orthohantavirus* replication cycle. Despite the diversity in the infection cycle of many negative stranded RNA viruses, we are hopeful that there are common mechanisms that can be targeted by anti-viral inhibitors. As it is challenging to develop drugs against each of these viruses, antiviral agents with broad spectrum antiviral activity could be the prime strategy to cope with this challenge [17].

## Methods

### DNA constructs and preparation of RNA standards

**Fig. 2:**
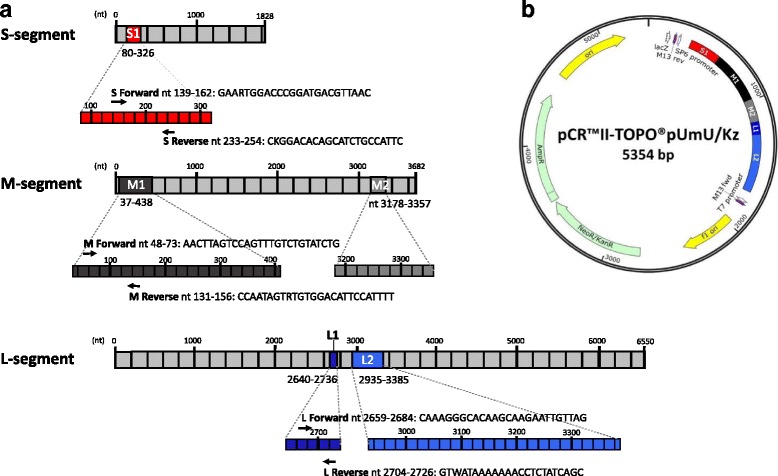
Genetic organization indicating the selected nucleotides of the S, M and L-segments of Puumala *Orthohantavirus * used for in vitro transcription and qRT-PCR. Fig. 2**a**. The five colored boxes (S1, M1, M2, L1 and L2), indicate the regions of the three segments that were fused and cloned side-by-side as synthetic DNA into the plasmid pCR™II-TOPO®pUmU/Kz. Below each box, the primers and the location in the corresponding RNA transcripts are shown. Fig. 2**b**. The chosen regions of the different segments are color-coded in red (S-segment), black (M-segment) and blue (L-segment) according, to their origin and positions in the corresponding viral RNA of Puumala *Orthohantavirus*, and visulized by using SnapGene software(GSL Biotech, Chicago, IL)

Sequences from PUUV strain Umea/hu, [18, 19] and PUUV strain Kazan [20] containing the S-segment nucleotides 80–326; M-segment (nt 37–438 and 3178–3357) and L-segment (nt 2640–2736 and 2935–3385), (Fig. 2a) were produced by Eurofins, MWG Operon, Germany, (Additional file 1: Figure S1). The above primer binding regions of the three segments were selected as target sequences for primers, and cloned side-by-side into the pCR™II-TOPO® vector (Invitrogen, Carlsbad, CA, US) resulting in the plasmid pCR™II-TOPO®pUmU/Kz, (Fig. 2b).

Primer pairs were designed and evaluated against the above sequences, and pairs giving the lowest Ct value against the corresponding S-, M- and L- regions are shown in Table 1. The primer pairs were designed with the aim of having approximately the same melting temperature (Tm), and minimizing primer dimer formation and self-annealing loops (cut-off values of − 5 kcal/mol for the Gibbs free energy [ΔG] of oligonucleotide interactions). Primer dimer interactions were investigated using Oligo Analyser 3.0 online tool (Integrated DNA Technologies, Inc.) and the predicted Tm and oligonucleotide interactions were found using the internet based software OligoCalc [21]. The selected primers were finally used for quantitative real-time PCR (qRT-PCR) of PUUV strain Kazan infected cells.

**Table 1:** Primers towards the S, M and L segments of PUUV

Segment	Primer name	Sequence	Binding
S-Primer	PuKzSF2	GAARTGGACCCGGATGACGTTAAC	139–162
	PuKzSR2	CKGGACACAGCATCTGCCATTC	233–254
M-Primer	PuKzMF1	AACTTAGTCCAGTTTGTCTGTATCTG	48–73
	PuKzMR1	CCAATAGTRTGTGGACATTCCATTTT	131–156
L-Primer	PuKzLF1	CAAAGGGCACAAGCAAGAATTGTTAG	2659–2684
	PuKzLR3	GTWATAAAAAAACCTCTATCAGC	2704–2726

The plasmid pCR™II-TOPO®pUmU/Kz containing equal proportions of the primer binding sites of the S-, M- and L-segments of PUUV strain Umea/hu and PUUV strain Kazan virus was linearized using EcoRV, downstream the SP6 promoter and the insert, and subsequently used for RNA transcription by the Riboprobe Combination System SP6/T7 RNA Polymerase (Promega, Wisconsin, USA), essentially as described before [22]. Finally, the DNA was removed after transcription with RNase free DNase, as specified in the protocol for Riboprobe Combination System SP6/T7 RNA Polymerase, and the concentration of the produced RNA was determined using a NanoDrop® ND-1000 Spectrophotometer (Saveen Werner, Malmö, Sweden). The RNA copy numbers were calculated from the obtained results, and diluted to a fixed concentration (10^10^ copies per mL) before storage at − 80 °C. Standardized amounts of in-vitro produced RNA was used in combination the S, M and L primers and qRT-PCR to produce three standard curves, one for each segment sequence. The obtained standard curves were finally used to calculate the copy numbers of S-, M- and L-segments in samples of infected cells.

### Cell culture, infections and RNA isolation

Twenty-four well plates were seeded with 170,000 cells per well (Vero E6, ATCC® CRL-1586). The cells were maintained in Dulbecco’s Modified Eagle Medium (DMEM, Sigma-Aldrich, St. Louis, MO, USA) containing 4.5 g/L D-glucose, 20 mM HEPES, 5% Fetal Bovine Serum (FBS), 0.75 g/L sodium bicarbonate and penicillin streptomycin at 37 °C, in a humidified atmosphere containing 5% CO_2_. The following day PUUV strain Kazan virus was diluted in serum free DMEM and used to infect cells at a multiplicity of infection (MOI) of 10, in a total volume of 200 μL in each well. After 2 h incubation at 37 °C, the media was removed and the cells were washed four times with 1 mL serum free DMEM per well before 1 mL DMEM containing 2% FBS was added. At indicated time points the supernatants and the carefully washed cells were collected separately and kept frozen at − 80 °C until analysis. The cells from individual wells were lysed in 350 μL RA1 Lysis Buffer (Macherey-Nagel GmbH & Co. KG, Düren, Germany) containing 3.5 μL β-mercaptoethanol. Total RNA was extracted and purified from cell lysates using a Nucleospin RNA kit (Macherey-Nagel GmbH & Co. KG) and from cell culture media using QIAamp Viral RNA Mini Kit (Qiagen, Hilden, Germany) according to the instruction of the manufacturer. The RNA of each sample was collected in 60 μL RNase free water.

### PCR conditions

The RNA expression levels of S-, M- and L-segments were quantified by qRT-PCR (StepOnePlus Real-Time PCR System, Applied Biosystems, Foster City, CA, USA) using the primers described in Table 1. The qRT-PCR was carried out in multiplate® PCR plates™ 96-well, clear wells (Bio-Rad Laboratories, Hercules, Ca, USA Inc.) containing 2 μL RNA, 200 nM of each primer, 12 μL of KAPA master mix components (KAPA SYBR® Fast Universal One-Step qRT-PCR Kit, KAPA Biosystems, Boston, MA, USA), and water to a final volume of 20 μL. The PCR cycling condition consisted of an initial 5 min incubation at 42 °C, for cDNA synthesis and 5 min at 95 °C for enzyme activation. This was followed by 40 cycles, each consisting of a denaturation step at 95 °C, for 3 s and an annealing/extension step at 60 °C for 25 s.

## Results

**Fig. 3:**
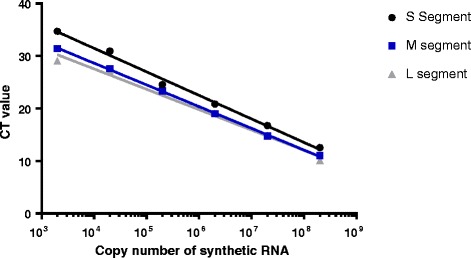
Standard curves. The graphs show the linear relation between the PCR cycles (CT value) and the calculated copy numbers of the in vitro produced RNA of the synthetic gene

Using a selection of primer pairs (Table 1), designed against the different PUUV RNAs, and predetermined copy numbers of in-vitro transcribed RNA, three amplification curves were produced, one from each of the S-, M- and L-segment primer pairs, (Fig. 3). The three nearly parallel curves, indicated a similar efficiency of amplification, from about 2000 copies of M- and L-transcripts and S transcripts to 2 × 10^8^ copies, when using the in-vitro produced RNA and the S-, M- and L-segment specific primer pairs.

**Fig. 4:**
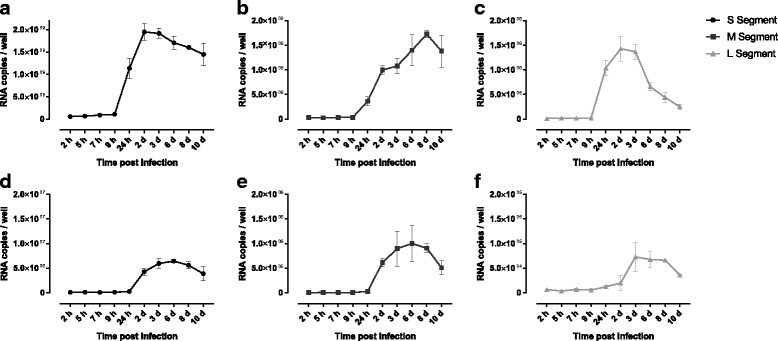
Copy numbers of Puumala *Orthohantavirus* RNAs’ at different time points post infection. Fig. 4**a**-**c** show the RNA levels of the S-, M and L segments, respectively, in samples (100 μL) prepared from Vero E6 cells, while the Fig. 4**d**-**f** show the corresponding samples from 100 μL cell culture media. The graphs show the mean average with standard deviation/error (SD). The samples were done in triplicates for each time-point and the experiment and the qPCR was repeated twice. The calculations were made from a total of four individual CT-values per time-point

When the kinetics of viral RNA production was analysed in Vero E6 cells infected with a MOI of 10 with PUUV strain Kazan, and the copy numbers of each of the three RNAs at different time points post infection was determined by qRT-PCR, the results indicated an increase in viral RNA production 9 h post infection. We noticed that the maximum copy number of transcripts from the S-segment and L-segments was found at day 2 with close to 2 billion (1.95 × 10^9^) copies/mL (S-segment) and nearly 1.5 million (1.43 × 10^6^) copies/mL (L-segment), with a decline after 2–3 days (Fig. 4).

**Fig. 5:**
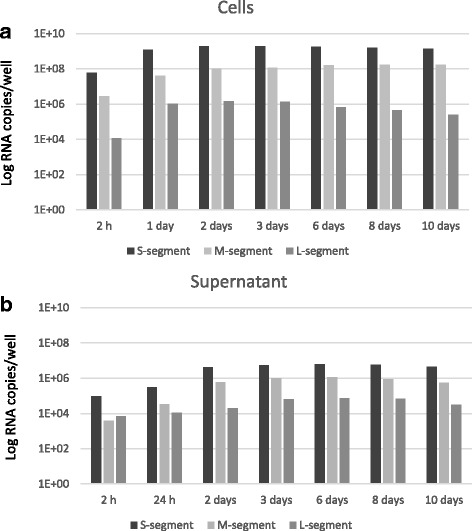
RNA copy numbers of the S-, M- and L-segments at different time points post infection. The black, the light gray and dark gray bars indicate the copy number of RNA originating from the S-, M- and L-segment, respectively. Fig 5**a** show the RNA levels in samples (100 μL) prepared from Vero E6 cells, while Fig. 5**b** show the corresponding samples from 100 μL cell culture media

The M transcripts reached its maximum (1.72 × 10^8^ copies/mL) later, at day 8. Interestingly, in contrast to the rapid appearance and decline of the L transcripts, the RNA produced from the S- and M-segments showed a prolonged period of transcription, (Fig. 4a-c). At peak days, the transcripts of the S- and M-segments became thousand and hundred-fold more abundant than transcripts of the L-segment, respectively (Figs. 4 and 5).

The RNA content in the supernatant of the infected cells was also analysed in parallel with the samples of the cells to follow the secretion of virions into the cell culture media. As shown by the graphs, a lower quantity of viral RNA copies was detected in the cell culture media during the timeframe of the experiments, in comparison to the corresponding samples of the cells. These results indicated a similar trend as observed from the infected cells, but the increase in number of viral RNA copies in the cell culture media became apparent later, after 24 h of post infection, (Fig. 4d-f).

## Discussion

The kinetics of viral replication specifically *Orthohantaviruses* needs to be investigated further, and there is lack of published data on the virus replication mechanisms that can support our current study.

In this study, an investigation of the kinetics and levels of individual RNAs of PUUV strain Kazan was conducted after synchronous infection of Vero E6 cells, as a marker for *Orthohantavirus* replication. Our results showed a rapid increase in viral RNA production in all samples of infected cells. Interestingly, the transcripts of the S- and M-segments became more abundant than transcripts of the L-segment both within the cell and in the cell supernatant. However, the viral RNA in supernatants were in general at lower levels. Virions with at least one S, M and L segment will be able to produce progeny virions upon infection, but we do not know the frequency of complete *Orthohantavirus* virions from our study. On the other hand, co-infection with complementing particles may result in productive infection, and for another 3-segmented negative sense RNA virus, Rift Valley fever virus, the majority of produced virions lacked one or more genome segments [23].

The differences in the kinetics and levels of transcription/replication may reflect the requirement of viral components during the synthesis and release of new virus particles. As an example, the delayed and prolonged viral RNA production from the M-segment, in comparison to the other segments, might reflect the need for the glycoproteins at later stages of viral replication. Thereafter, the transcription/replication of the different viral RNAs stagnated at different copy numbers and decreased thereafter. The reduction of RNA copy numbers, after reaching the plateau values, could be the result of cell death and release of nucleases resulting in degradation of viral RNA. This post transcriptional degradation may influence the observed levels and ratios of the different RNAs, both in the cell lysates and RNA in the cell culture media.

We believe that our key findings could provide a basis for a better understanding of the replication kinetics of viral RNA transcripts across the different segments of *Orthohantavirus*. This may aid in the search for useful therapeutic compounds which may yield to better patient management and novel treatments.

## Conclusions

We have developed a method to follow *Orthohantavirus* transcription/replication in-vitro after synchronous infection of Vero cells. By using our method we can monitor the production and levels of the individual viral RNAs of the RNPs during an ongoing infection.

## Additional file

Additional file 1: Figure S1. The nucleotide sequence of the synthetic DNA. The selected sequence (1377 bp) are color coded to show the origin of the sequences in the RNA segments of Puumala *Orthohantavirus*. The nucleotides 80–326, shown in red letters, originate from the S segment; the black letters indicate the nucleotides 37–438 and 3178–3357 of the M-segment while the blue letters indicate the nucleotides 2640–2736 and 2935–3385 of the L-segment. (PPTM 34 kb)

## Abbreviations

Gn and Gc: Amino- and carboxy-terminal portions of the polyprotein
HCPS: Hantavirus cardiopulmonary syndrome
HFRS: Hemorrhagic fever with renal syndrome
Kz: Puumala *Orthohantavirus* strain Kazan
MOI: Multiplicity of infection
N protein: Nucleocapsid protein
nt: Nucleotide
PBMC: Peripheral blood mono-nucleated cells
PUUV: Puumala *Orthohantavirus*
qRT-PCR: Quantitative real-time PCR
RdRp: RNA-dependent RNA polymerase
RNP: Ribonucleoproteins
UmU: Puumala *Orthohantavirus* strain Umea/hu
