# Supplementary material for: Quantification and kinetics of viral RNA transcripts produced in Orthohantavirus infected cells
Source: Virol J. 2018 Jan 19;15:18. doi: 10.1186/s12985-018-0932-8 (PMC5775559; doi:10.1186/s12985-018-0932-8)
Supplement: Additional file 1: Figure S1. — The nucleotide sequence of the synthetic DNA. The selected sequence (1377 bp) are color coded to show the origin of the sequences in the RNA segments of Puumala Orthohantavirus. The nucleotides 80–326, shown in red letters, originate from the S segment; the black letters indicate the nucleotides 37–438 and 3178–3357 of the M-segment while the blue letters indicate the nucleotides 2640–2736 and 2935–3385 of the L-segment. (PPTM 34 kb) [file 12985_2018_932_MOESM1_ESM.pptm]

## Slide 1
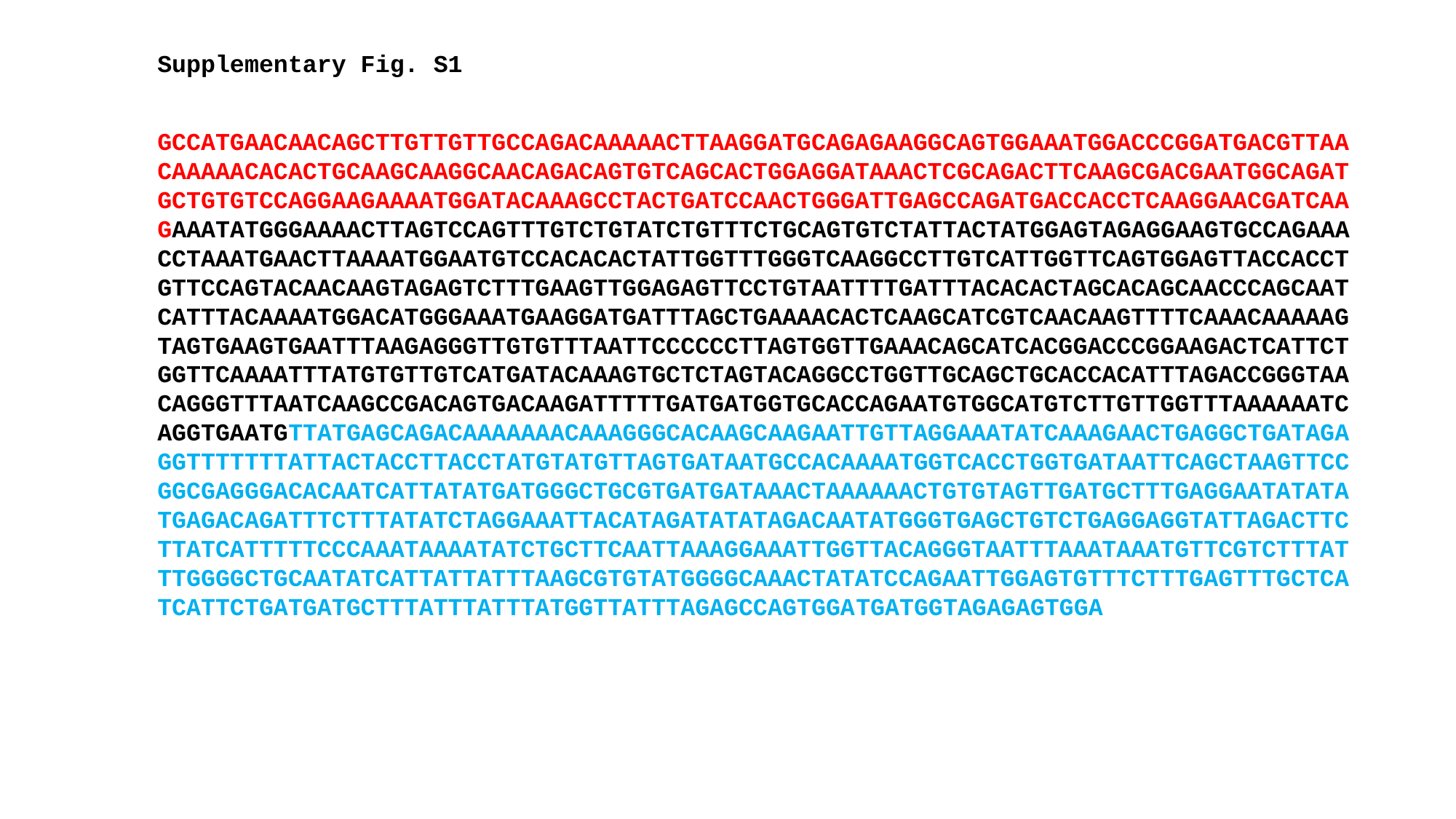

Supplementary Fig. S1
GCCATGAACAACAGCTTGTTGTTGCCAGACAAAAACTTAAGGATGCAGAGAAGGCAGTGGAAATGGACCCGGATGACGTTAACAAAAACACACTGCAAGCAAGGCAACAGACAGTGTCAGCACTGGAGGATAAACTCGCAGACTTCAAGCGACGAATGGCAGATGCTGTGTCCAGGAAGAAAATGGATACAAAGCCTACTGATCCAACTGGGATTGAGCCAGATGACCACCTCAAGGAACGATCAAGAAATATGGGAAAACTTAGTCCAGTTTGTCTGTATCTGTTTCTGCAGTGTCTATTACTATGGAGTAGAGGAAGTGCCAGAAACCTAAATGAACTTAAAATGGAATGTCCACACACTATTGGTTTGGGTCAAGGCCTTGTCATTGGTTCAGTGGAGTTACCACCTGTTCCAGTACAACAAGTAGAGTCTTTGAAGTTGGAGAGTTCCTGTAATTTTGATTTACACACTAGCACAGCAACCCAGCAATCATTTACAAAATGGACATGGGAAATGAAGGATGATTTAGCTGAAAACACTCAAGCATCGTCAACAAGTTTTCAAACAAAAAGTAGTGAAGTGAATTTAAGAGGGTTGTGTTTAATTCCCCCCTTAGTGGTTGAAACAGCATCACGGACCCGGAAGACTCATTCTGGTTCAAAATTTATGTGTTGTCATGATACAAAGTGCTCTAGTACAGGCCTGGTTGCAGCTGCACCACATTTAGACCGGGTAACAGGGTTTAATCAAGCCGACAGTGACAAGATTTTTGATGATGGTGCACCAGAATGTGGCATGTCTTGTTGGTTTAAAAAATCAGGTGAATGTTATGAGCAGACAAAAAAACAAAGGGCACAAGCAAGAATTGTTAGGAAATATCAAAGAACTGAGGCTGATAGAGGTTTTTTTATTACTACCTTACCTATGTATGTTAGTGATAATGCCACAAAATGGTCACCTGGTGATAATTCAGCTAAGTTCCGGCGAGGGACACAATCATTATATGATGGGCTGCGTGATGATAAACTAAAAAACTGTGTAGTTGATGCTTTGAGGAATATATATGAGACAGATTTCTTTATATCTAGGAAATTACATAGATATATAGACAATATGGGTGAGCTGTCTGAGGAGGTATTAGACTTCTTATCATTTTTCCCAAATAAAATATCTGCTTCAATTAAAGGAAATTGGTTACAGGGTAATTTAAATAAATGTTCGTCTTTATTTGGGGCTGCAATATCATTATTATTTAAGCGTGTATGGGGCAAACTATATCCAGAATTGGAGTGTTTCTTTGAGTTTGCTCATCATTCTGATGATGCTTTATTTATTTATGGTTATTTAGAGCCAGTGGATGATGGTAGAGAGTGGA
